# Multi-epitope design against emerging nipah virus towards peptide vaccine development

**DOI:** 10.6026/973206300223786

**Published:** 2026-06-30

**Authors:** Motukuri Naveen Kumar, Dokka Muni Kumar, Bharathi B.V.N.V., Vamseedhar Annam, Aarthi Pradhan, Shaik Rajiya Sulthana, Yashasvi Singam

**Affiliations:** 1 Department of Life Sciences, School of Allied and Healthcare Sciences, Malla Reddy University, Hyderabad, Telangana, India; 2 Department of Pathology, Sapthagiri Institute of Medical Sciences and Research Center, Bangalore, India; 3 Department of Microbiology, Naran Lala College of Professional and Applied Sciences, Veer Narmad South Gujarat University, Surat, Gujarat, India

**Keywords:** Nipah virus (NiV), multi-epitope vaccine, immunoinformatics, reverse vaccinology, phosphoprotein, in silico analysis, immune simulation

## Abstract

The lack of an approved vaccine against Nipah virus (NiV), a highly fatal zoonotic pathogen causing severe neurological and respiratory disease, remains a major global health challenge. Therefore, it is of interest to design a multi-epitope vaccine targeting the NiV phosphoprotein (UniProt ID: Q9IK91). Conserved B-cell, cytotoxic T-lymphocyte (CTL) and helper T-lymphocyte (HTL) epitopes were identified and selected based on antigenicity, immunogenicity and safety. The resulting vaccine construct was evaluated through structural modeling, TLR4 docking, codon optimization and immune simulation analyses. Thus, data shows the favorable stability, strong receptor interaction, efficient expression potential and the ability to elicit robust humoral and cellular immune responses.

## Background:

Computational vaccine design utilizes immunoinformatics and structural bioinformatics to rapidly identify and optimize epitope-based candidates, offering a faster and more cost-effective strategy for addressing emerging viral threats [1]. Emerging infectious diseases remain a major threat to global health, often driven by novel zoonotic pathogens and exacerbated by environmental changes and increased human-wildlife interaction [2]. Among these, Nipah virus (NiV), a highly pathogenic paramyxovirus, presents a serious risk due to its high fatality rate (40-75%), lack of licensed therapies and frequent outbreaks in South and Southeast Asia [3, 4]. NiV, classified as a Biosafety Level 4 (BSL-4) pathogen, can cause acute respiratory distress, encephalitis and multi-organ failure [5]. Human-to-human transmission, combined with high mutation rates, increases its potential for adaptation and global spread [6]. Despite the public health burden, there are no approved vaccines or antivirals, underscoring the urgent need for novel preventive strategies. Traditional vaccine platforms (live-attenuated, inactivated, subunit, vector-based) face safety, efficacy and manufacturing constraints. Multiple candidates (viral vectors, VLPs, mRNA, DNA) targeting G and F glycoproteins are under study, but none are licensed [7, 8]. Recent advances in computational biology have enabled the rapid, cost-effective development of peptide-based multi-epitope vaccines using immunoinformatics tools [9]. NiV proteins G, F and N, known for their immunogenicity and conserved epitopes, serve as ideal targets [10]. The first documented occurrence of the Nipah virus (NiV) was identified in 1998 in the Malaysian region of Sungai Nipah, subsequently followed by reports from Bangladesh, India, Singapore and the Philippines [11]. Both homosapiens and variety of animal spaces demonstrate susceptibility to a wide array pathologies caused by NiV, which includes conditions that range from mild to severe encephalitis, as well as potentially lethal respiratory syndromes [12]. Moreover the availability of natural nipah virus isolate sequences its notable limited thereby restricting the inferences that can be regarding the evolutionary pressures suggested by sequence variability [13]. Therefore, it is of interest to report the in silico design of a multi-epitope vaccine targeting the nipah virus phosphoprotein.

## Materials and Methods:

## Retrieval of nipah virus protein sequences: 

The complete protein sequences of the Nipah virus (NiV) were retrieved from the National Center for Biotechnology Information (NCBI) and the Universal Protein Resource (UniProt) databases, referencing the primary accession number Q9IK91 (https://www.uniprot.org/uniprotkb/Q9IK91/entry). The glycoprotein (G), fusion protein (F) and nucleocapsid protein (N) were chosen as primary targets due to their critical roles in viral entry, fusion and evasion of the host immune system. Sequence alignment was performed using Clustal Omega to identify conserved regions across various Nipah virus strains.

## Prediction of antigenic peptides:

The antigenic peptides that are likely to be antigenic by eliciting antibody response antigenic peptides are predicted following the method of Kolaskar and Tongaonkar [14]. Predictions are based on a table that reflects the occurrence of amino acid residues in experimentally known segmental epitopes.

## Identification of B-cell epitopes:

B-cell epitopes were predicted using the ABCpred server, which employs recurrent neural networks to identify linear epitopes based on the input amino acid sequence. A fixed-length prediction method was used and epitopes were ranked by their scores, with higher scores indicating greater epitope potential.

## Identification of T-cell epitopes:

HTL epitopes play a crucial role in prophylactic and immunotherapeutic vaccines. IEDB MHC-I epitope prediction tool (http://tools.iedb.org/mhci/) was used for HTL prediction and the MHC-II tool (http://tools.iedb.org/mhcii/) for class MHC-II cells. Default parameters for allele selection were set to H@Ab and H2_1ad. The results are ranked based on binding affinity, with lower ranks indicating stronger binding to the HTL receptor.

## Determination of antigenicity and allergenicity:

Shortlisted epitopes were evaluated for antigenicity using VaxiJen v2.0, considering scores above 0.45 as probable antigens. Allergenicity was assessed using AllerTOP v2.0, which classifies proteins via the k-nearest neighbour (kNN, k=1) algorithm based on a dataset of known allergens and non-allergens.

## Construction of multi-epitope vaccine:

The final multi-epitope vaccine construct was designed by linking the selected B-cell and T-cell epitopes using appropriate linkers. A potent immunostimulatory adjuvant, such as TLR agonists, is essential for effective activation of both innate and adaptive immunity. The adjuvant was linked to epitopes using EAACK linkage. A total of four linkers were used to construct the final vaccine - EAAAK, GGGGS, GPGPG and KK. EAAAK was linked to the adjuvant sequence, GGGGS to the cytotoxic T lymphocyte (CTL) epitopes, GPGPG to the helper T lymphocyte (HTL) epitopes and KK to the B-cell lymphocyte epitopes.

## Prediction of physicochemical properties of the vaccine:

Various physicochemical parameters were assessed using the online web server ProtParam (http://web.expasy.org/protparam/).

## Prediction of secondary structure:

Secondary structure prediction was performed using the PSIPRED server (http://bioinf.cs.ucl.ac.uk/psipred/). The accuracy of the predictions is improved by integrating two feed-forward neural networks (ref), which simultaneously evaluate the outputs from the initial network and analyze the input derived from preliminary predictions generated by PSI BLAST (Position Specific Iterated BLAST).

## Prediction of Tertiary structure:

The construct's tertiary structure was predicted using the I-TASSER server, which identifies PDB templates via LOMETS and performs iterative fragment assembly to generate atomic models. Five 3D models were produced, with functional insights derived from BioLip-based re- threading.

## Molecular docking:

The ClusPro v2 server (https://cluspro.org/login.php) was used to evaluate the interaction between the vaccine construct and TLR4. The refined model of the vaccine construct, as the ligand and TLR4, as the receptor, were submitted to the server.

## Immune simulation:

The C-ImmSim server was used to simulate immune responses to the final vaccine construct. It models immunological memory and lymphocyte proliferation using predictive algorithms, including PSSM and evaluates molecular binding via Miyazawa-Jernigan potentials.

## Codon optimisation and in silico cloning of the final vaccine construct:

Reverse translation and codon optimization were carried out using the Java Codon Adaptation Tool (JCat) server (http://www.prodoric.de/JCat) to express the multi-epitope vaccine construct in a selected expression vector. Codon optimization targeted expression in E. coli strain K12. The JCat output includes the codon adaptation index (CAI) and GC content percentage, which assess protein expression levels. A CAI score close to 1.0 is ideal, with scores above 0.8 considered good. The GC content should range between 30-70%, as values outside this range may negatively impact translational and transcriptional efficiencies.

## Results:

The complete protein sequences of the Nipah virus (NiV) were retrieved from publicly available repositories, including the National Center for Biotechnology Information (NCBI) and the Universal Protein Resource (UniProt) databases. The Nipah virus phosphoprotein comprises 709 amino acid residues, as detailed in the complete protein sequence retrieved from the UniProt database under accession number Q9IK91 which can be accessed through the UniProt database at https://www.uniprot.org/uniprotkb/Q9IK91/entry. Antigenic peptides were predicted using the method of Kolaskar and Tongaonkar [14]. The 709-residue Nipah virus phosphoprotein showed an average antigenic propensity score of 1.0027. A total of 25 antigenic peptide regions were predicted across the protein sequence, indicating the presence of multiple potential immune-reactive sites (Table 1). The prediction method used for this analysis has a reported accuracy of approximately 75%, ensuring a reasonable level of reliability while acknowledging some margin for error. The Immune Complex (IC) plot for the Nipah virus phosphoprotein (Q9IK91) was generated to identify potential B-cell epitopes (Figure 1 - see PDF). It mapped antigenic propensity across the 709-amino acid sequence, highlighting regions with elevated scores suggestive of immunogenic potential. These results offer insights into the protein's antigenic landscape, aiding vaccine design and immunological research. The B-cell epitopes of the Nipah virus phosphoprotein were identified using the Kolaskar and Tongaonkar [14] antigenicity prediction method. Totally 34 epitopes were identified by using ABDpred prediction server where default threshold was set at 0.51. The predicted B cell epitopes are ranked according to their score obtained by trained recurrent neural networks. Higher score of the peptide means the higher probability to be an epitope. Out of 34, 3 B-cell epitopes were selected basing on score obtained by recurrent neural network for the vaccine construct (Table 2). The identification of MHC class I-restricted cytotoxic T lymphocyte (CTL) epitopes was performed using silico prediction tools, which evaluated epitope binding affinity, immunogenicity and antigenicity. Candidate epitopes were screened based on their binding strength to multiple HLA alleles, proteasomal processing efficiency and TAP transport potential. Following a comprehensive analysis, three high-affinity MHC class I epitopes was selected for inclusion in the vaccine construct (Table 3). These epitopes demonstrated strong immunogenic potential and stability, ensuring their ability to elicit a robust CTL-mediated immune response and their non-allergenic characteristics, supports their suitability for eliciting a robust CTL-mediated immune response. The selection of MHC class II-restricted helper T-cell (HTL) epitopes was conducted using *in silico* prediction tools, evaluating their binding affinity to multiple HLA-DR alleles, antigenicity, immunogenicity and conservancy. To ensure vaccine safety, allergenicity assessments were performed and only non-allergenic epitopes were considered. Following a stringent screening process, three high-affinities, antigenic and non-allergenic MHC class II epitopes were selected for inclusion in the vaccine construct (Table 4). These epitopes exhibited strong interactions with HLA-DR molecules, facilitating robust CD4^+^ T-cell activation. A total of three B-cell epitopes, three CTL epitopes and three HTL epitopes were merged to construct the multi-epitope vaccine (Figure 2 - see PDF). Four linkers - EAAAK, GGGGS, GPGPG and KK - were utilized in the construction of the final vaccine. The EAAAK linker was used to fuse the adjuvant sequence, ensuring structural stability and efficient immune stimulation. The GGGGS linker was incorporated between cytotoxic T lymphocyte (CTL) epitopes to maintain flexibility and epitope presentation. The GPGPG linker was employed to separate helper T lymphocyte (HTL) epitopes, enhancing immune processing and recognition. Lastly, the KK linker was used to connect B-cell epitopes, optimizing antigenic exposure for effective humoral immune response induction. A sequence of 159 amino acids was generated after epitope fusion.

(MAKLSTDELLDAFKEMTLLELSDFVKKFEETFEVTAAAPVAVAAAGAAPAGAAVEAAEEQSEFDVILEAAGDKKIGVIKVVREIVSGLGLKEAKDLVDGAPKPLLEKVAKEAADEAKAKLEAAGATVTVK) was added to the N-terminal of the vaccine sequence by an EAAAK linker. The final designed vaccine construct consisted of 323 amino acids. The 323-amino acid long peptide sequence containing an adjuvant (L7/L12 ribosomal protein - blue) at the amino terminal end linked with the multi-epitope sequence through an EAAAK linker. B epitopes and HTL epitopes are linked using GPGPG linkers (green) while the CTL epitopes are linked with the help of GGGGS linkers (red). The secondary structure of the final subunit vaccine model was analyzed, revealing that 28% of the structure is composed of alpha helices, 4% is made up of beta strands and 47% is classified as disordered regions (Figure 3 - see PDF). This suggests that the vaccine construct has a substantial portion of flexible, disordered conformations, with a smaller proportion of well- defined secondary structures such as alpha helices and beta strands. This structural distribution may influence the vaccine's stability and immunogenicity. The 3D model of the multi-epitope vaccine construct was evaluated with a C-score of -3.15, indicating moderate prediction quality (Figure 4 - see PDF). The estimated TM-score of 0.36 ± 0.12 suggests low to moderate similarity to the native structure, while the estimated RMSD of 14.0 ± 3.9 Å indicates moderate deviation from experimental structures. These results suggest the model may require further refinement for improved accuracy. The Ramachandran plot analysis of the 3D structure of the multi-epitope vaccine construct revealed that 76.0% of the residues fall within the core region, indicating optimal backbone geometry (Figure 5 - see PDF). 18.2% of the residues are in the allowed region and 3.1% are in the generously allowed region, suggesting minimal steric clashes. Small percentages, 2.7%, of residues were found in the disallowed region, which may require further investigation. The analysis of side-chain parameters showed 41 labelled residues out of 321 and 17 labelled residues out of 157 for chi-square plots. Additionally, the maximum deviation of 19.2 was noted and bond length/angle deviations were recorded at 7.5. The presence of bad contacts was identified, but these did not significantly affect the overall structure. G-factors for dihedral and covalent bond parameters were -0.97 and -0.36, respectively, indicating reasonable quality, with an overall G-factor of -0.68, suggesting a moderately favourable structure. Regarding planar groups, 82.5% were within limits, while 17.5% were highlighted as potentially problematic and may warrant further investigation. Overall, the Ramachandran plot analysis indicates a structurally reliable model with areas that may benefit from refinement. The quality of the 3D structural model of the vaccine construct was assessed using the ERRAT tool, which evaluates non-bonded atomic interactions to detect structural errors. The model achieved an overall quality factor of 92.381, indicating high reliability and accuracy (Figure 6 - see PDF). This result confirms the construct's suitability for further immunological and biochemical analyses. The 3D structural quality of the multi-epitope vaccine construct was assessed using validation tools; resulting in both an average score and a raw score (Figure 7 - see PDF). The average score reflects the overall structural integrity, while the raw score highlights specific regions for potential refinement. Both scores indicated a high-quality structure, suitable for further applications, with any suboptimal areas identified for further improvement. The molecular docking between the vaccine construct and TLR4 was conducted using the ClusPro 2.0 server to examine the interaction between the vaccine construct and the TLR4 receptor (Figure 8 - see PDF). A total 30 models were generated and among them, only that model was selected which properly occupied the receptor and having lowest energy score and found that model number 0.00 fulfil the desired criteria that's why selected as the best-docked complex. The energy score obtained for the model 0.00 was found to be 691.5 which are lowest among all other predicted docked complex showing highest binding affinity. The docking analysis suggests that the vaccine construct forms stable interactions with the TLR4 receptor's A-chain, potentially triggering immune responses through activation of this key pattern recognition receptor. The color-coded chains of TLR4 and the vaccine construct in red provide a clear visual representation of their interaction at the molecular level, crucial for understanding the mechanism of immune activation and the vaccine's role in eliciting a protective immune response. The coding sequence of the multi-epitope vaccine construct was optimized to match the codon usage bias of *Escherichia coli BL21 (DE3)* for efficient expression. Rare codons were replaced with synonymous ones frequently used in *E. coli,* enhancing translational efficiency and protein yield. Optimization also accounted for GC content, mRNA stability and avoidance of undesirable motifs. The optimized sequence achieved a codon adaptation index (CAI) near 1.0, ensuring compatibility with the host system for robust vaccine production. The GC content of the multi-epitope vaccine construct was analyzed to assess its suitability for expression in *Escherichia coli BL21 (DE3).* Before optimization, the GC content was 00.00%, which was suboptimal for the bacterial expression system, potentially affecting mRNA stability and translational efficiency. After codon optimization, the GC content was adjusted to 55.42%, achieving a balanced composition suitable for efficient transcription and translation in the host. This optimization enhances mRNA stability and ensures high-yield protein expression (Figure 9 - see PDF). The multi-epitope vaccine construct was engineered using the pET-6xHis/ {TAA} vector, a bacterial protein expression vector optimized for high-efficiency recombinant protein production (Figure 10 - see PDF). The vector spans 6629 base pairs (bp) and incorporates a 6xHis tag, facilitating affinity purification of the recombinant protein through binding to nickel or cobalt-based resins. This construct was expressed in *Escherichia coli BL21 (DE3) cells,* which are specifically engineered for high-yield expression of recombinant proteins via the T7 RNA polymerase system, inducible by IPTG. For the cloning process, the VB Ultra stable host was utilized to enhance plasmid stability, ensuring robust propagation and maintenance of the construct. This combination of a stable cloning system and an efficient expression host ensures optimal protein yield and stability for further vaccine development and immunological studies. Figure 11 (see PDF) illustrates the simulated immune response to the chimeric peptide vaccine, generated using C-ImmSim, an advanced *in silico* immune system simulator. The figure visualizes the population dynamics of key immune cells over 35-day period post-vaccination, providing insights into immune activation, proliferation and regulatory mechanisms. The simulation tracks variations in cytotoxic T cells, natural killer cells, macrophages, dendritic cells and epithelial cells, offering a comprehensive representation of the adaptive and innate immune responses. Cytotoxic T cell activation is evident in the early phase, followed by a decline that coincides with an increase in anergic cells. NK cells peak during the initial immune response, consistent with their role in early pathogen clearance. The antigen-presenting function of macrophages and dendritic cells is sustained, supporting the induction of adaptive immunity. These trends help elucidate the temporal progression of immune activation, antigen presentation and potential immune regulation following vaccine administration. The simulated dynamics of B-cell and T-helper (TH) cell populations following immunization with the chimeric peptide vaccine, modeled using C-ImmSim, provide a comprehensive representation of adaptive immune responses over a 35-day period (Figure 12 - see PDF). The graphs illustrate key immunological mechanisms, including the expansion and contraction of B-cell populations, memory B-cell formation and the kinetics of antibody production. Similarly, the TH cell population dynamics capture activation, differentiation and memory formation, highlighting their role in orchestrating immune responses through cytokine signaling and antigen-specific activation. This simulation offers valuable insights into the progression of immune memory, the development of long-term immunity and the interplay between cellular and humoral immune responses post-vaccination. C-IMMSIM simulation indicates the concentration of cytokines and interleukins, with the inset plot displaying the danger signal alongside the leukocyte growth factor IL-2 (Figure 13 - see PDF). The inset plot also shows the IL-2 level, with the Simpson index (D) marked by the dotted line. D represents a measure of diversity, where an increase in D over time signifies the emergence of different epitope-specific dominant T-cell clones. A smaller D value indicates lower diversity. The antigen clearance curve shows rapid antigen decline post-vaccination, reflecting an effective immune response (Figure 14 - see PDF). This figure depicts the temporal dynamics of antigen levels and immunoglobulin (Ig) responses over a 35-day period following immunization. The simulation, conducted using C-ImmSim, showcases the effectiveness of antigen clearance and the progression of the humoral immune response. The initial IgM peak, followed by sustained IgG production, indicates successful class switching. The dominance of IgG1 suggests TH1-biased immune activation, enhancing cellular immunity for effective clearance of intracellular pathogens. As it supports the activation of mechanisms that target and eliminate infected cells. Therefore, the observed pattern of antibody production and antigen clearance confirms a well-coordinated and efficient immune response, tailored to handle specific types of infections.

## Vaccines construct sequence:

EAAAKMAKLSTDELLDAFKEMTLLELSDFVKKFEETFEVTAAAPVAVAAAGAAPAGAAVEAAEEQSEFDVILEAAGDKKIGVIKVVREIVSGLGLKEAKDLVDGAPKPLLEKVAKEAADEAKAKLEAAGATVTVKE AAAKAKFVAAWTLKAAAGGGGSREDLILPELGGGGSKSRGIPIKKGGGGSMPKSRGIPIGPGPGNVQLNASTAVKETDKGPGPGLNYHADHLGGPGPGLPELNFEETNASQFVKKKGKGERKGKNNPELKPKKSS PERGWSDYTSGANNKKHVRGSPPYQEGKSVNAKKAKFVAAWTLKAAAGGGGS

Linkers- EAAAK, GGGGS, GPGPG, KK, PADRE sequence

## Discussion:

Vaccine development for emerging pathogens like Nipah virus (NiV) is critical due to its high mortality and outbreak potential. This study employs *in silico* immunoinformatics to design a multi-epitope vaccine, offering a rapid, targeted alternative to traditional methods. Computational tools enable identification of highly immunogenic epitopes without pathogen cultivation, accelerating development [15]. Multi-epitope vaccines show promise for clinical translation, combining enhanced antigenicity, reduced off-target effects and scalability for effective viral protection [16]. The phosphoprotein of the Nipah virus (NiV), consisting of 709 amino acid residues (UniProt accession number Q9IK91), is central to the virus's replication and pathogenesis, making it an attractive target for immunological research and vaccine development [17]. This protein is involved in the virus's transcription and replication processes and interacts with host cell machinery, which underscores its potential as a vaccine target. The detailed structural and functional characterization of this protein is essential for the identification of antigenic determinants that could serve as vaccine epitopes. Antigenic peptides from the NiV phosphoprotein were predicted using the Kolaskar and Tongaonkar method [14], a well-established approach for antigenicity prediction. This analysis identified 25 potential antigenic determinants with an average antigenic propensity score of 1.0027, indicating a slight above- baseline probability for these regions to elicit an immune response. The prediction accuracy, estimated at approximately 75%, is based on known antigenic patterns, providing a reliable framework for selecting candidate epitopes for immunization [18]. Further validation through the Immune Complex (IC) plot demonstrated potential B-cell epitopes within the NiV phosphoprotein, which are key targets for humoral immunity. Previous studies have successfully identified critical B-cell epitopes for various viral pathogens, reinforcing the utility of computational approaches in vaccine development [19]. Refining the analysis, the ABDpred server was employed to identify 34 B-cell epitopes based on their predicted scores [20]. The top three epitopes, with the highest predicted immunogenic scores, were selected for inclusion in the vaccine construct. This strategy aligns with approaches used in the development of vaccines targeting other viral pathogens, such as Ebola [21], ensuring that the most immunogenic regions are prioritized for vaccine design. In parallel, T-cell epitope prediction was performed to design a vaccine capable of eliciting a robust cellular immune response. MHC class I-restricted cytotoxic T lymphocyte (CTL) epitopes and MHC class II-restricted helper T lymphocyte (HTL) epitopes were predicted, focusing on their binding affinities to various HLA alleles. These epitopes were selected based on their high immunogenic potential, stability and non-allergenic properties [22]. Ensuring that the vaccine could provoke a comprehensive immune response involving both cellular and humoral immunity [23]. The multi-epitope vaccine construct was assembled by fusing three B-cell epitopes, three CTL epitopes and three HTL epitopes using four different linkers. The use of flexible linkers, such as EAAAK and GGGGS, is a common approach in multi-epitope vaccine design, as they provide structural flexibility and promote optimal epitope presentation [24]. The resulting construct is designed to stimulate a broad-spectrum immune response by targeting multiple facets of immunity, as evidenced by the success of similar multi-epitope vaccines in combating other viral infections [1]. Structural analysis showed that 47% of the vaccine construct is disordered, typical of flexible peptides that may enhance antigenic exposure and immune receptor interaction. The 3D model, with a C-score of -3.15, indicates moderate reliability, requiring refinement for improved stability. The Ramachandran plot, with 76% of residues in favorable regions, supports the construct's structural integrity for effective immunogenicity. Molecular docking studies further validated the proposed vaccine by showing strong binding interactions between the epitopes and immune receptors, particularly Toll-like receptor 4 (TLR-4). Molecular docking simulations of the vaccine construct with the TLR4 receptor showed strong interactions, with a docking score of -691.5, indicating the potential for activating the TLR4 signaling pathway, a critical component of the innate immune response [26]. The docking results revealed that the multi-epitope vaccine construct forms stable complexes with TLR-4, which could enhance the innate immune response and act as a crucial first step in activating adaptive immunity. Molecular dynamics simulations confirmed the stability of vaccine-receptor interactions, supporting the construct's structural integrity. Consistent with previous studies on multi-epitope vaccines for Zika and Dengue [27], the stable docking model indicates strong binding affinity, suggesting the vaccine can effectively activate innate immune receptors and elicit a potent immune response. Codon optimization of the vaccine sequence for expression in *Escherichia coli BL21 (DE3)* was performed to maximize translational efficiency and protein yield. The codon adaptation index (CAI) was optimized to a value close to 1.0, ensuring compatibility with the host expression system and facilitating efficient production of the vaccine. Additionally, optimization of GC content further enhanced mRNA stability, which is crucial for high-level protein expression. *In silico* immune simulations (C-ImmSim) predicted a robust yet regulated response to the chimeric peptide vaccine. The transition from active to anergic T cells indicates immune control to prevent excessive inflammation. Antigen-presenting cells (APCs) like dendritic cells and macrophages support antigen processing for long- term memory. The vaccine's epithelial stability minimizes cytotoxicity, enhancing safety. It primes both humoral and cellular immunity, with immunoglobulin class switching and anergic T/B cells suggesting tolerance. Broad activation of TH1, TH2 and TH17 subsets promotes effective clearance and memory. IgM-to-IgG switching and IgG1 dominance indicate strong, TH1-mediated protection against intracellular pathogens. Immune simulations predict strong immune responses and memory cell formation, supporting long-term immunity against NiV. The balanced TH1/ TH2 response prevents immunopathology and the population coverage analysis, based on high-affinity HLA-binding epitopes, ensures broad effectiveness across diverse populations, aligning with previous studies [28]. The simulations indicated activation of cytotoxic T-cells and natural killer cells, with sustained activity of antigen-presenting cells, such as macrophages and dendritic cells, confirming the capacity of the vaccine to induce a coordinated and long-lasting immune response. Using immunoinformatics, a multi-epitope vaccine targeting MHC-I, MHC-II and B-cell epitopes from NiV glycoprotein and nucleocapsid protein was designed and applied reverse vaccinology to identify suitable Nipah virus epitopes and construct a multi-epitope vaccine candidate with encouraging immunogenic properties [29]. Computational simulations confirmed its stability, strong TLR binding and immune activation potential. Codon optimization and *in silico* cloning indicated high expression in *E. coli.* While promising, further *in vitro* and *in vivo* validation is needed to confirm efficacy. The traditional vaccine development process is resource- intensive and time-consuming, requiring pathogen cultivation in laboratory settings [30]. In contrast, reverse vaccinology leverages computational analysis and genomic data to design vaccines without the need for pathogen cultivation. Key steps in this approach include the prediction of T and B cell epitopes, antigen processing, antigenicity, allergenicity, toxicity assessments and TLR-peptide docking, all of which are essential for designing effective virus vaccines [31]. A multi-epitope vaccine with promising antigenic and immunogenic properties using computational epitope prediction methods has been reported [32]. Recent studies have also demonstrated the growing potential of computational vaccine design for Nipah virus. Multi-epitope vaccine candidates have been shown to possess strong immunogenic potential and the ability to generate broad immune responses against the virus [33]. The integration of machine learning with immunoinformatics has further improved the identification of highly immunogenic epitopes and the development of structurally stable vaccine constructs [34]. Vaccine designs based on NiV structural proteins have also been predicted to elicit both humoral and cellular immune responses, highlighting their potential for effective immune protection [35]. Additionally, several multi-epitope vaccine constructs have exhibited favorable antigenicity, safety, and stability profiles, underscoring the value of reverse vaccinology as a powerful approach for the rapid development of Nipah virus vaccines [36].

## Conclusion:

We show that the Nipah virus phosphoprotein as a suitable candidate for epitope-based vaccine design. The proposed multi-epitope vaccine showed encouraging antigenic and immunological features in computational assessments. These findings provide preliminary evidence for its potential and warrant further experimental evaluation.

## Figures and Tables

**Table 1:** Prediction of antigenic peptides of nipah virus phosphoprotein

**n**	**Start Position**	**Sequence**	**End Position**
1	46	EDFLQCT	52
2	103	DDIQILDPVVTDVVYH	117
3	145	NGNVCLVSD	153
4	155	KMLSYAPEIAV	165
5	172	TDLVHLE	178
6	187	NPTAVPFTL	195
7	203	KDSPVIAEHYYGLGVKE	219
8	231	NLDSIKL	237
9	257	SSSEVIVGI	265
10	321	PQKRLPM	327
11	360	AQPPYHW	366
12	379	IVNGAVQT	386
13	427	ATRHVRGSPP	436
14	444	NAENVQLNASTA	455
15	496	LNYHADH	502
16	507	DLETLCEESVLMGVINSIKLI	527
17	536	IEEQVKEIPKIINK	549
18	552	SIDRVLA	558
19	563	ALSTIEGHLVSM	574
20	593	ELKPVIGR	600
21	602	IEEQQSLFSF	611
22	627	YGAAVQLREDLILPE	641
23	649	ASQFVPM	655
24	660	SRDVIKTLIR	669
25	678	RSELIGY	684

**Table 2:** Choice of B-cell Epitopes for vaccine construct

**RANK**	**SEQUENCE**	**START POSITION**	**SCORE**
1	KGKGERKGKNNPELKP		
		581	0.95
2	SSPERGWSDYTSGANN		
		130	0.94
3	HVRGSPPYQEGKSVNA	430	0.93

**Table 3:** MHC class I-restricted cytotoxic T lymphocyte (CTL) epitopes

**HLA type**	**Sequence position**	**Peptide**	**Allergen and non-allergen**
HLA-B*40:01	634	REDLILPEL	Antigen,
			non-allergen
HLA-A*30:01	398	KSRGIPIKK	Antigen,
			non-allergen
HLA-B*07:02	396	MPKSRGIPI	Antigen,
			non-allergen

**Table 4:** MHC class II-restricted helper T-cell (HTL) epitopes

**HLA type**	**Sequence position**	**Peptide**	**Allergen and non-allergen**
HLA-DRB1*09:01	447	NVQLNASTAVKETDK	Antigen, non-allergen
HLA-DQA1*04:01/DQB 1*04:02	493	LNYHADHLG	Antigen, non-allergen
HLA-DRB1*04:01	639	LPELNFEETNASQFV	Antigen, non-allergen
